# Intergenerational consequences of violence: violence during pregnancy as a risk factor for infection in infancy

**DOI:** 10.3389/fgwh.2024.1397194

**Published:** 2024-07-12

**Authors:** Lukas Blumrich, Braian Lucas Aguiar Sousa, Marco Antônio Barbieri, Vanda Maria Ferreira Simões, Antonio Augusto Moura da Silva, Heloisa Bettiol, Alexandre Archanjo Ferraro

**Affiliations:** ^1^ Department of Pediatrics, Faculty of Medicine, University of São Paulo, São Paulo, Brazil; ^2^ Department of Pediatrics, Ribeirão Preto Medical School, University of São Paulo, Ribeirão Preto, Brazil; ^3^ Department of Public Health, Federal University of Maranhão, São Luís, Brazil

**Keywords:** domestic violence, intimate partner violence, gender-based violence, infection, infancy

## Abstract

**Introduction:**

Psychosocial stress during pregnancy has long-lasting and important consequences in the following generations, as it can affect intrauterine development. The impact on the developing immune system is notoriously important due to the associated morbidity and mortality in the first years of life. Little attention has been given to the role of violence during pregnancy (VDP), especially its impact on infant infectious morbidity.

**Methods:**

We analyzed data from two Brazilian birth cohorts (*n* = 2,847) in two distinct cities (Ribeirão Preto and São Luís), collected during pregnancy and at the beginning of the second year of life. The association between VDP and infection in infancy was analyzed with structural equation modeling, using the WHO-VAW questionnaire as exposure and a latent variable for infection as the outcome.

**Results:**

VDP was reported by 2.48% (sexual), 11.56% (physical), and 45.90% (psychological) of the mothers. The models presented an adequate fit. In the city of São Luís, VDP was significantly associated with the latent construct for infection (standardized beta = 0.182; *p* = 0.022), while that was not the case for the Ribeirão Preto sample (standardized beta = 0.113; *p* = 0.113). Further analyses showed a gradient effect for the different dimensions of the exposure, from psychological to physical and sexual violence.

**Conclusion:**

Our results suggest an association of VDP with infant morbidity in a poorer socioeconomic setting, and highlight the importance of considering the different dimensions of intimate partner violence. These findings may have important implications for the comprehension of global health inequalities and of the effects of gender-based violence.

## Introduction

Maternal psychosocial stress during pregnancy has far-reaching effects on future generations, impacting both immediate and long-term health (1, 2). It affects approximately one-quarter of pregnant women globally (3) and is associated with a wide range of consequences for offspring's health, encompassing perinatal complications as well as long-term conditions including cardiovascular disease, metabolic syndrome, and neuropsychiatric disorders (4, 5).

Stress during pregnancy is a multifaceted concept that encompasses various factors, including depression or anxiety, perceived stress, natural disasters, heat stress, and experiences of discrimination. Although violence during pregnancy is a well-known global issue, it has received relatively less attention as a specific stressor. Given the variety of consequences of intimate partner violence, such as depression, higher perceived stress, and social isolation, it is imperative to investigate its distinct effects to fully comprehend the extent of the negative consequences associated with gender-based violence (6).

Studying violence during pregnancy presents numerous challenges, including obtaining reliable data and discerning its specific impacts. Additionally, the influence of environmental factors, such as socioeconomic status (3, 7) and culture (8, 9) (e.g., perceptions of intimate partner violence within relationship dynamics) remains unclear. Considering the complexity of a phenomenon such as intimate partner violence, it is likely that the impacts of violence vary across different populations (10, 11).

The wide-ranging consequences of maternal violence on offspring should not be underestimated. Extensive evidence indicates its association with maternal morbidity during (12–14) and after (14, 15) pregnancy, premature birth (16, 17), low birth weight (16–18), intrauterine growth restriction (19), undernutrition (20), infectious morbidity (21, 22), delayed neurodevelopment (23), and higher mortality (24). More recently, some studies have investigated the effects of prenatal violence on infectious morbidity in the offspring. Most of the studies that sought to clarify this relation used national surveys and evaluated the association between the lifetime occurrence of violence and specific morbid conditions in children at variable ages (22, 25–29).

To the best of our knowledge, the investigation of violence during pregnancy and its effects on infant morbidity of offspring in humans was done by only one study (30). Expanding these investigations is important to adequately comprehend the extent of the negative effects of violence, and to identify potential intervention targets for offspring morbidity and mortality.

There are a number of possible biological pathways for psychosocial stress and violence to impact on the development of the immune system, such as disruption of corticosteroid dynamics (31, 32), impaired transference of passive immunity, and changes in the gut microbiome (33). The first one could give rise to a suppressed immune response in the infant (34, 35) and disrupt thymus development (36), while changes in placental antibody transfer (37) could be deleterious at the beginning of the infant's life. Since the gut microbiota participates in the regulation of inflammatory processes, its disruption by stress could be detrimental, and stress during pregnancy may even influence the whole composition of the offspring's own intestinal microbiota (33, 37).

This study sought to investigate the association of violence during pregnancy with infant infectious morbidity in two Brazilian birth cohorts from cities with markedly different socioeconomic contexts.

## Materials and methods

### Study design

This is an observational study using data from the cohort “Etiological Factors of Preterm Birth and Consequences of Perinatal Factors for Child Health: Brazilian Birth Cohort Study of Ribeirão Preto and São Luís (BRISA)” (38).

### Population and sample

We analyzed the data from the birth cohorts of the cities Ribeirão Preto and São Luís, collected at three distinct waves (prenatal, perinatal, and postnatal, during the second year of infants’ life). The BRISA cohort study began data collection in 2010.

Ribeirão Preto is a wealthy and industrialized city in the State of São Paulo, with a Human Development Index (HDI) of 0.800, 604,682 inhabitants, of which 2.53% are below the poverty line, and a per capita income of USD 730.02 as of 201 0 (39). More than 99% of the residences receive piped water and more than 98% are equipped with sanitary sewage (40). São Luís is the capital of the State of Maranhão, in one of the poorest regions in the country, with a HDI of 0.768, 1.014.837 inhabitants, 13.81% below the poverty line, and a per capita income of USD 447.42 (39). 82% of residents have access to piped water, while only 45.7% were equipped with sanitary sewage as of 2010 (40).

A convenience sample was used in both cities. Pregnant women were identified in hospitals and health units on the occasion of a prenatal visit, and women with singleton pregnancies who had undergone an obstetrical ultrasound during the first trimester of gestation were invited to participate. The sample of São Luís consisted of 1.447 pregnant women, interviewed initially, from February 2010 to June 2011, while Ribeirão Preto had 1.400 subjects, interviewed between February 2010 and February 2011.

Interviews were held between the 22nd and the 25th week of pregnancy by a previously trained team to collect data on reproductive health, demographic and socioeconomic status, details of pregnancy, and life habits of the mothers. In Ribeirão Preto, data were collected at the Clinical Research Unit of the University Hospital, University of São Paulo, while in São Luís data were collected at the Clinical Research Center of the Mother-Child Unit of the University Hospital in São Luís.

Immediately after childbirth (i.e., in the period of 24 h after birth), interviews were conducted by teams of trained collaborators in maternity homes in both cities, collecting information about mothers and babies using standardized questionnaires. All team members were trained to perform data collection.

A new evaluation was conducted at the beginning of the second year of the infant's life, with age ranging from 12 to 36 months (median age was 18 months). Interviews were again conducted at University Hospitals in both cities, with standardized questionnaires about child's health and medical events, as described below.

All phases of the study were approved by the Research Ethics Committee of the University Hospital of the Ribeirão Preto Medical School/University of São Paulo (protocol 4116/2008) and the University Hospital of Federal University of Maranhão (protocol 4771/2008-30). All mothers who participated in the project signed the free and informed consent form after reading and understanding the project's aim and methods. Trained professionals helped to clarify information on the consent form when needed.

The sample size for the city of Ribeirão Preto was 1,400, while the sample size for São Luís was 1,447 dyads.

### Variables

The chosen exposure was violence suffered during pregnancy, measured by the WHO Violence Against Women Questionnaire (41). We analyzed this data using a previously validated latent variable based on the first 13 questions of the questionnaire (42) (4 for the psychological, 6 for the physical, and 3 for the sexual dimension of violence).

The chosen outcome was built as a latent construct for the morbidity of the infant, derived from the following variables, obtained during the second-year interview with the mother: occurrence of fever in the last 2 weeks, occurrence of diarrhea in the last 2 weeks, and use of anti-inflammatory medication in the last 2 weeks, use of antibiotic medication in the last 2 weeks. The latent construct was analyzed as a continuous variable.

Additional variables, recognized as potential confounders, were also collected. These confounders were identified using a theoretical framework constructed using Direct Acyclic Graph Methodology (DAG) from the dagitty software (43). The identified confounders were: maternal age, marital situation, social support and support networks, maternal education, maternal occupation, and family income. The last three variables were used to build a latent construct for socioeconomic status, and added as a confounder in the model. Maternal age was analyzed as a continuous variable, while marital situation was categorized as married, living together, single or divorced/widowed. Social support and support networks were measured by the Medical Outcomes Study (MOS) Social Support survey (44), and analyzed as continuous (45). All of the instruments were administered in Brazilian Portuguese following transcultural adaptation, as reported in the original description of the birth cohort (38).

### Data analysis

Categorical variables were described using absolute and relative frequencies. Continuous variables were described using mean and standard deviation.

Structural equation modelling was performed in MPlus Software (46) using the Weighted Least Square Mean and Variance (WLSMV) estimator and theta parameterization, in order to obtain adequate control for residual differences in variances. Models results were standardized. We expected the following results for model fit, based on previous research (47, 48): a value of RMSEA (Root Mean Square Error of Approximation) <0.08, CFI (Comparative Fit Index) >= 0.90 and TLI (Tucker-Lewis Index) >= 0.95; and a WRMR (Weighted Root Mean Squared Residual) value of <1.0. Ideally, *p*-value estimates for model fit should be above a value of 0.05. Considering our sample sizes, however, this metric is not expected to be attained and does not importantly impact model fit when other parameters are adequate.

Data was analyzed separately for each city, as we believe they constitute different populations and one of the study’s goals was to evaluate the relationship between exposure and outcome in different socioeconomic contexts.

## Results

The structural equations models constructed with the latent variables and the confounders fitted the data adequately, as shown in Table 1. The Weighted Root Mean Squared Residuals (WRMR) for both models were higher than 1.0, but, since this index is experimental and the other indexes indicate a very good fit, both models were considered adequate.

**Table 1:** Model fit for the structural equation models of association between violence during pregnancy and fever in the last 2 weeks in two birth cohorts in distinct cities (Ribeirão Preto and São Luís).

Indexes	São Luís	Ribeirão Preto
χ^2^	1,422.233	1,520.531
Degrees of freedom	759	759
*p*	0.0000	0.0000
RMSEA	0.025	0.027
90% CI	0.023–0.027	0.025–0.029
*p*-value	1.000	1.000
CFI	0.990	0.993
TLI	0.990	0.992
WRMR	1.291	1.391

χ^2^, chi squared test; RMSEA, root mean square error of approximation; 90% CI, 90% confidence interval; CFI, comparative fit index; TLI, Tucker Lewis Index; WRMR, weighted root mean square residual.

The descriptive statistics presented in Table 2 show important differences between the two cohorts. Maternal age was very similar for both cities, but the São Luís sample had almost two times as many non-skilled workers as Ribeirão Preto (19,70% vs. 10,40%, *p* < 0.001) and lower income (*p* < 0.001). Maternal educational level in São Luís saw more women completing the secondary level, but tertiary levels were very similar between the cities. As for marital status, Ribeirão Preto saw a higher rate of formally married women, but both cities had similar frequencies of single or divorced/widowed women.

**Table 2:** Description of the sample characteristics of two birth cohorts from Brazilian cities [São Luís (*n* = 1,447) and Ribeirão Preto (*n* = 1,400)] in 2010.

Variable	Descriptive Statistics					
	Category	Frequency (%)			*P*-Test	
Exposure		Total (*n* = 2,847)	São Luís (*n* = 1,447)	Ribeirão Preto (*n* = 1,400)	*T*-Test	Chi Square
Psychological violence	–	45.90	48.41	43.24	–	0.006
Physical violence	–	11.56	12.40	10.67		0.151
Sexual violence	–	2.48	2.84	2.11		0.216
Outcomes						
Fever^a^	–	8.39	7.95	8.86	–	0.382
Diarrhea^a^		13.91	14.58	13.21	–	0.292
Antibiotic medication^a^		5.09	3.52	6.72	–	<0.000
Anti–inflammatory medication^a^		13.66	12.72	14.64	–	0.135
Covariates						
Maternal age	Mean	25.76	25.75	25.76	0.936	–
Householder's occupation	Does not apply	11.28	11.13	11.43	–	<0.001
	Manual and non-skilled	27.18	24.63	29.84	–	–
	Manual and skilled	45.56	43.18	48.03	–	–
	Non manual and non-skilled	11.06	14.49	7.50	–	–
	Non manual and skilled	4.08	5.21	2.90	–	–
	Not active	0.84	1.36	0.30	–	–
Marital status	Married	29.16	22.74	35.82	–	<0.001
	Living together	51.42	57.43	45.20	–	–
	Single	17.59	18.04	17.12	–	–
	divorced/widowed	1.83	1.80	1.86	–	–
Family's income	<1 MS	4.68	4.99	4.35	–	<0.001
	1–3 MS	52.30	56.05	48.14		
	3–5 MS	42.94	38.82	47.51		
	5 or more	0.07	0.14	0.00		
Mother's educational level	Primary school	20.05	12.53	27.83	–	<0.001
	Secondary school	69.87	75.55	63.99	–	–
	Tertiary incomplete	5.53	7.34	3.66	–	–
	Tertiary complete	4.55	4.57	4.52	–	–

MS, minimum salary.

^a^ In the last 2 weeks.

As for the violence exposure during pregnancy, mothers in São Luís reported psychological violence (as understood as any affirmative response in the items related to psychological violence in the WHO questionnaire) more frequently than mothers in Ribeirão Preto (48.41% vs. 43.24%, *p* = 0.006). Fever in the last 2 weeks has a similar frequency in both cities, of 7.95% and 8.86%, (*p* = 0.382), respectively. Diarrhea saw a similar distribution (*p* = 0.292). While in São Luís 3.52% of the mothers reported at the use of antibiotic medication two weeks prior to the interview, in Ribeirão Preto this rate was 6.72% (*p* < 0.001), and the use of anti-inflammatory medication was similar in both cities.

Our analysis show (Table 3) that violence during pregnancy was associated with infant morbidity in the city of São Luís (standardized beta = 0.182, *p* = 0.022) but not in the city of Ribeirão Preto (stardardized beta = 0.113, *p* = 0.113). In the city of São Luís, given the results found, we conducted a post-hoc analysis for each dimension of violence (psychological, physical, and sexual) separately as the exposure (Table 4). Effect sizes tended to increase as it would be expected in the case of a gradient effect (psychological violence beta = 0.053, *p* = 0.405; physical violence beta = 0.115, *p* = 0.220; sexual violence beta = 0.428, *p* < 0.001), but only the sexual violence was found to be statistically significantly associated with infant morbidity.

**Table 3:** Standardized model results (STDYX) for the latent variable for infection on latent variable for violence.

City	Standardized estimate (Beta)	Standard error	*p*-value
Ribeirão Preto	0.113	0.071	0.113
São Luís	0.182	0.083	0.022

**Table 4:** Standardized model results for the regression of the latent variable for infection different levels of the latent variable for violence in the city of São luis.

Independent latent construct	Estimate	Standard error	*p*-value
Violence	0.182	0.083	0.022
Psychological	0.053	0.064	0.405
Physical	0.115	0.094	0.220
Sexual	0.428	0.105	0.000

## Discussion

Our results suggest a context-specific effect for violence during pregnancy on infant morbidity, as measured by fever, diarrhea, or use of antibiotic or anti-inflammatory medication in the last two weeks. This association was present in the city of São Luís, but could not be detected in the city of Ribeirão Preto, and saw a gradient effect with increasing effect sizes when considering the psychological, physical, and sexual dimensions of violence.

Considering the distinct socioeconomic characteristics of the two cities, the impact of violence during pregnancy was observed exclusively within the population living in the city with lower socioeconomic status. São Luís, in comparison to Ribeirão Preto, displayed significantly lower levels of human development, as evidenced by a notably lower Human Development Index (HDI) score (0.768, ranking 249th in Brazil) compared to Ribeirão Preto's higher HDI score (0.800, ranking 40th). Additionally, São Luís exhibited lower per capita income (USD 447.42) (39) compared to Ribeirão Preto (USD 730.02) (40) at the time of data collection.

Albeit some statistically significant differences could be noted between the two samples (Table 1), the samples share very similar characteristics overall and the São Luis sample presented higher levels of maternal education. This holds substantial implications, as it suggests a systemic or contextual effect for socioeconomic status (SES). Considering the similarities in the SES of both samples, the finding limited to the city of São Luís can be plausibly explained by the differences in health service quality and access to adequate healthcare and education between the two cities. The suggestion that some negative effects of pregnancy exposures may only be detected in harsher systemic socioeconomic conditions can contribute to a better understanding of health disparities and challenges, both prevalent in the Global South.

This context-specific effect may explain the results of Manzolli et al. (30), that found no association between prenatal exposure to violence and diarrhea or respiratory infection in infancy. This study, the only other one to evaluate the effects of violence during pregnancy on infant morbidity, was conducted in the southern region of Brazil. The socioeconomic conditions in the cities where data was collected for their study are much more similar to Ribeirão Preto than to São Luís, and thus are in line with our findings.

The presence of a gradient effect observed across various dimensions of violence, as assessed using the WHO instrument, provides compelling evidence supporting the association under investigation. While no statistically significant association was found when considering psychological and physical violence individually, the statistically significant findings obtained when considering all dimensions collectively may suggest insufficient statistical power due to the inclusion of many confounding variables with complex interrelationships in our structural equation models.

Notably, a substantial standardized effect size was observed for sexual violence, emphasizing the significance of incorporating this dimension of violence in analyses. To the best of our knowledge, no prior studies have specifically examined the effects of sexual violence during pregnancy on longer term offspring heath (i.e., later than the neonatal period). Considering how common forced intercourse is worldwide (49) and how sexual violence is a sexual manifestation of gender-based violence (50), our findings underscore the importance of including this dimension in future research to comprehensively understand the complete range of detrimental effects resulting from violence.

The biological mechanisms possibly responsible for this association are not completely understood. A well-stablished hypothesis concerns the importance of cortisol in fetal development (31, 32). The 11β-HSD2 enzyme in the placenta has a protective function, converting most of the maternal cortisol that reaches fetal circulation into the inactive cortisone (51). Stressed mothers, however, in addition to presenting higher levels of circulating cortisol, may have impaired function of this enzyme, leading to a higher exposure of the fetus to cortisol during development (32, 37). This exposure in turn may modulate immune system development and function, mainly through programming of the hypothalamic-pituitary-adrenocortical axis of the fetus (32, 51). It was also previously suggested that the diminished function of the mother's immune system due to exposure to higher levels of glucocorticoids may impair the transplacental transfer of passive immunity (32). Another proposed mechanism suggests the role of the microbial gut on the development of the immune function of the offspring, since altered patterns of colonization are related to maternal stress during pregnancy and these may have a range of consequences (33, 52).

It is also important to consider how the experience of violence can be an obstacle for access to adequate healthcare (53). Women who experience intimate partner violence are less likely to attend adequate prenatal care, and may be less prone to search for healthcare in case of illness (53, 54). Households with the experience of violence, for this reason, may be chronically at a higher risk for morbidity. The first observation may induce a selection bias in our study, since mothers were recruited at a prenatal care appointment. Since in the Brazilian healthcare system there is an active strategy for screening and following up pregnant women, however, we believe this possible bias does not significantly impact our findings. Having less access to healthcare in general, however, is another potential explanation for increased offspring morbidity. Mothers who experience high levels of stress may also have impaired ability to perceive children's worsened health conditions, and, since our outcomes were based on mothers’ recall ability, this could underestimate the effect size we found. There is no available research on this phenomenon, and it remains an assumption.

**Figure 1:**
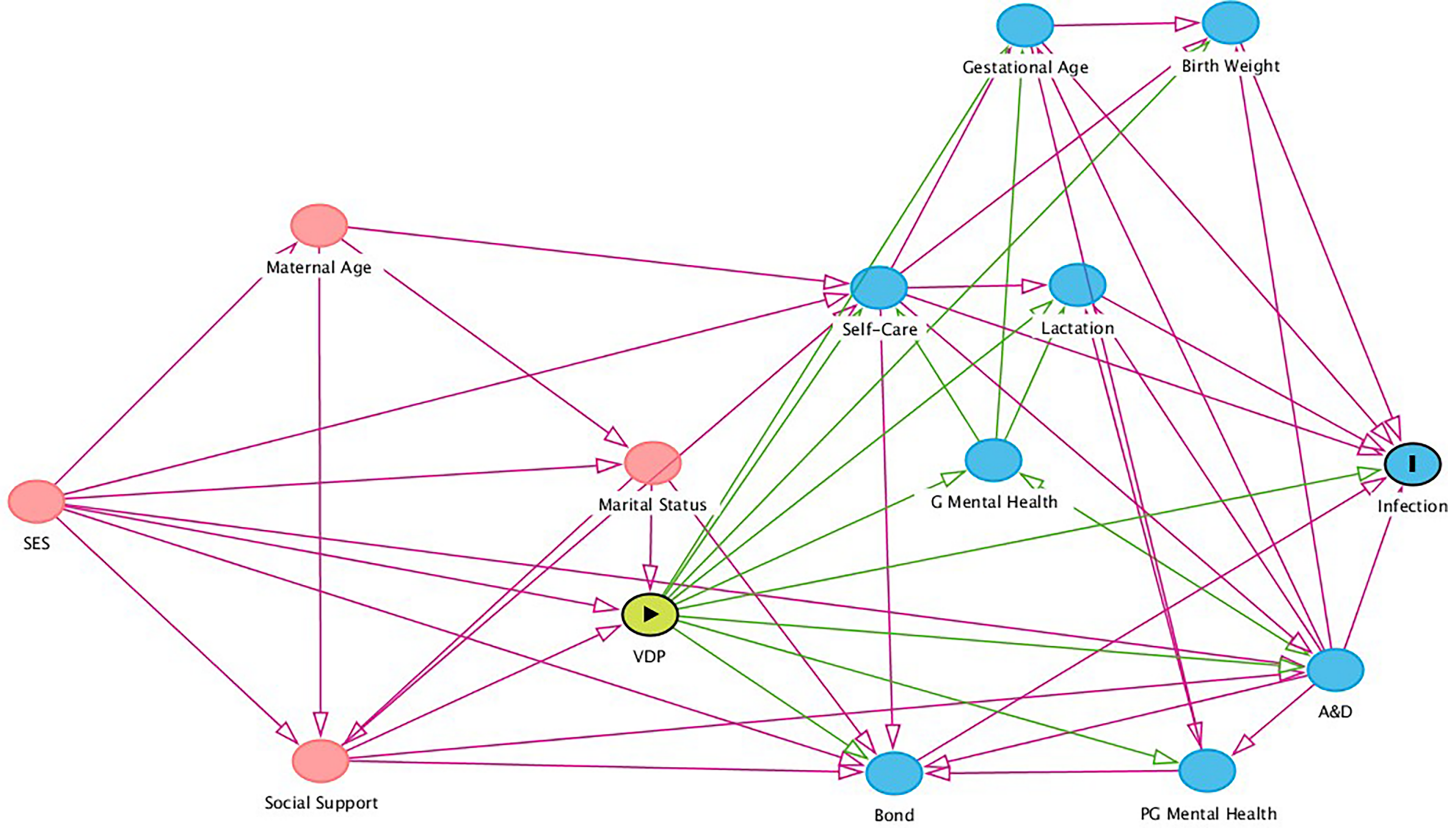
Directed acyclic graph (DAG) depicting the epidemiological model for the association between violence during pregnancy and fever in infancy. SES, Socioeconomic Status; VDP, Violence During Pregnancy; G Mental Health, Mother's Mental Health during Pregnancy; A&D, Alcohol and Drugs use during Pregnancy; Bond, Maternal-infant Bonding; PG Mental Health, Mother's Mental Health Post Pregnancy. Variables in red are ancestral variables of both exposure and outcome, and thus are recognized as confounders in the theoretical model. Variables in blue are recognized as mediators of the relationship between exposure and outcome in the model.

Considering the theoretical model constructed for this analysis (Figure 1), we believe there is also the possibility of a behavioral pathway that may be partially responsible for the association here analyzed. The violence during pregnancy may impair a woman's ability to adequately care for herself as manifested in, for example, not attending healthcare appointments or searching for help when in need (54, 55). Violence during pregnancy may also impair the quality of the bond between mother and offspring, and it is reported that breastfeeding may be importantly affected by the experience of violence (56–58). The same applies for maternal mental health during and after pregnancy and substance use (58–61). All these factors may be directly related to child's health, and thus may act as mediators (62) of this relationship.

Our findings highlight the importance of thorough screening for violence against pregnant women and the importance of a gender and equity-based perspective in prenatal care. The impact of VDP for the infants’ health has already been established (6), but our findings may shed light on outcomes previously ignored. It may also add more weight to the benefits of violence screening recommendations (5, 63, 64). Efforts to mitigate the impact of violence should be made, such as devoting more time to understand each patient's context and discussing which are the possibilities for social and legal support (65, 66). The pathways through which it acts shall be the object of future studies, but as we understand it as a psychosocial stressor, already established strategies may be useful, such as group therapy, referral to social service professionals, empowering women to recognize and seek their rights and resources available to face situations of violence (66).

The strengths of our study include the use of the DAG methodology and the inclusion of a diversity of confounders in our models. Considering the array of confounders identified with the DAG methodology and included in the model, there is hardly an unmeasured confounder that could explain away this association. All the data was collected using validated instruments and using a standardized methodology. Our sample included populations from markedly different socioeconomic and cultural backgrounds, but who share the same language and political system, and thus comparisons are possible.

Some limitations should be noted. The first and most important is the underestimation of the violence suffered by the subjects included, mostly due to fear of retaliation or judgment. To minimize this possibility, all the interviewers were received previous training directed at collecting information about violence. The second is the memory bias present in the data here analyzed, as the sole source of information for each variable was the interviewed mother. For this reason, we chose to use a latent variable as the outcome for this analysis, since the variables used for its construction present complementary information on child morbidity. Lastly, the methodology used for sampling does not allow the generalization of results, but provides very strong internal validity for the study of the mechanisms of disease.

## Conclusion

The results here presented suggest an important association of violence during pregnancy and infant morbidity in the second year of life in a harsher socioeconomic setting. Moreover, a gradient effect for the different dimensions of violence was observed. Considering the ubiquity of violence against women, these findings may have important implications for public health and for the comprehension of global health inequalities. Furthermore, they underscore the pressing need for comprehensive efforts to address gender-based violence on a global scale.

## Data Availability

The datasets presented in this article are not readily available because the data here analyzed is the property of the RPS Consortium, and questions or requests can be directed to the corresponding author for the correct referral. Requests to access the datasets should be directed to Lukas Blumrich, lukas.blumrich@fm.usp.br.
